# Benefits and Risks of the Technological Creep of LED Light Technologies Applied to the Purse Seine Fishery

**DOI:** 10.3390/biology11010048

**Published:** 2021-12-29

**Authors:** Pasquale Ricci, Nicola Trivellin, Daniela Cascione, Giulia Cipriano, Viviana Teresa Orlandi, Roberto Carlucci

**Affiliations:** 1 Department of Biology, University of Bari, Via E. Orabona 4, 70124 Bari, Italy; daniela.cascione@uniba.it (D.C.); giulia.cipriano@uniba.it (G.C.); roberto.carlucci@uniba.it (R.C.); 2 CoNISMa, Piazzale Flaminio 9, 00196 Rome, Italy; 3 Department of Industrial Engineering, University of Padova, Via Venezia 1, 35131 Padova, Italy; nicola.trivellin@unipd.it; 4 Department of Bioscience and Life Science, University of Insubria, Via J-H Dunant 3, 21100 Varese, Italy; viviana.orlandi@uninsubria.it

**Keywords:** anchovy, CO_2_ emissions, CPUE, fishery sustainability, light emitting diode (LED)

## Abstract

**Simple Summary:**

The purse seine (PS) fishery with lamps is one of the most effective fishing techniques in the Mediterranean Sea, targeting phototactic organisms, such as anchovies and sardines. However, the employment of high-power lamps for many hours to aggregate fish schools involves an increase in costs for fuel and negative consequences on the environment. In this study, the catch efficiency of LED light technology was compared to the traditional incandescent lamp employed in the PS fishery in the Adriatic Sea. Three LED lights (white, blue, and pulsing) were compared with the incandescent lamp for catch efficiency, energy and hourly fuel consumption, CO_2_ emissions, and economic costs. The white LED increased efficiency by more than 2 times per unit of energy and fuel consumption, while the pulsing LED and blue LED increased efficiency by about 6 and 9 times, respectively. The CO_2_ emissions were reduced by approximately 2 and 8 times with white and blue LEDs, respectively. The potential positive economic impacts derived from the LED technology on the PS fishery in terms of fuel cost-saving percentages were all higher than 60%. This technology shows the potential economic benefits for fishermen and the mitigation of negative effects on the environment.

**Abstract:**

This study is a first attempt to investigate the catch efficiency of LED light technology compared to the traditional incandescent lamp that is used in the purse seine fishery (PS) in the Central Adriatic Sea (Mediterranean Sea). Catches per unit effort were adopted to assess the performance of lighting systems, considering the electrical energy and the fuel consumption as effort units. Concerning the catch efficiency, the white LED, which emits the same light spectra as the incandescent lamp, increased the yield by over 2 times per consumption unit of energy and fuel. The yield efficiency increased up to approximately 6 and 9 times when adopting the pulsing white or blue LED, respectively. These increases were due to the energy savings resulting from the flashing of the white LED or by the greater water penetration of the blue LED. No significant difference in target species sizes was detected between the use of LEDs and the incandescent lamp. The results obtained from estimates of the hourly fuel consumption and CO_2_ emissions stress potential benefits in the reduction of the carbon footprint due to the use of LEDs within the PS fishery. Positive economic impacts were derived from the LED technology on the PS fishery, with the fuel cost-saving percentages all being higher than 60%. The LED technology clearly shows potential benefits at the economic level for the fishermen, and the possibility of mitigating indirect negative effects on the environment due to fuel combustion and greenhouse gas emissions. On the other hand, the application of new technology that improves the catch efficiency of fishing gears should be carefully considered. The lack of regulations controlling technological advancement could cause unwanted long-term effects.

## 1. Introduction

The sustainable management of fishing stocks is a fundamental point to maximize the benefits provided by the fishery, while aiming to reduce anthropic impacts on marine ecosystems, as well as to increase economic growth and social welfare [1,2,3,4,5]. In particular, the ecosystem-based fishery management approach (EBFM, [6]) requires a move from the traditional management strategy, focused on preventing species populations from declining, towards the conservation of ecosystems in a healthy, productive, and resilient condition so they can provide the services useful for human life. In this regard, it is necessary to consider additional elements in a holistic pathway, such as interactions with other species, the effects of environmental changes, or pollution and other stressors on habitat and water quality. The great challenge of this approach is due to the high complexity derived from interactions between environmental phenomena, technological aspects, and socio-economic problems, as well as the uncertainties in the assessment of stochastic processes related to the exploited populations. Therefore, the need to add the human dimension to environmental goals has become urgent in the EBFM approach, requiring the use of indicators able to assess the performance of the fishing exploitation technique, integrated with ecological, social, and economic aspects [7]. From this point of view, the performance of fishing gears is closely related to their catch efficiency and fishing effort, which have increased over time because of technological progress (or technological creep), becoming a critical node in sustainability-oriented fisheries management [8,9]. In fact, since the 1990s, a reconstruction of global catches has indicated a condition of decline, despite an increase in fishing effort and catch efficiency, with the consequence of causing overfishing conditions and unsustainable fisheries [10,11]. At the same time, the improvement in catch efficiency of fishing gears could represent a key element in moving the fishery towards a sustainable dimension. In this challenge, the building of knowledge on the performance of innovative fishing devices and regulations required for effective fishery management should be realized according to the EBFM approach [12].

Within the EBFM framework, the understanding of relationships between the technological advancement and the catch efficiency of fishing gears has become a priority [13], as well as the integrated assessment of negative impacts on fishing stocks and the environment, such as from fuel consumption and emission of greenhouse gases (GHG) [14,15]. In this research field, an exemplary case is represented by the purse seine (PS) fishery operating with lamps to gather positive phototactic organisms (fishes and cephalopods), which is one of the most effective fishing techniques at the global scale [16,17]. This fishing technique is characterized by the involvement of a main vessel equipped with a purse seine net and smaller boats with lamps to attract fish during the night [18,19,20]. High-power lamps illuminate the fishing zone for many hours, aggregating the schooling fish, which are then encircled by the purse seine net. Therefore, expensive costs in fuel are involved in providing energy for the lamps, with the consequence of not being sustainable for the environment [21,22]. Historically, this technique used incandescent, fluorescent, halogen, and metal halide lamps because of their high luminant efficiency [23]. Since the 2000s, in southeast Asian countries, light emitting diode (LED) technology has been applied to the PS fishery instead of traditional lamps [17]. The LED lamp is a type of semiconductor diode lamp with direct current, and can be illuminated in a variety of colours that emit incoherent monochromatic light when given a forward voltage [24]. LED performance in terms of environmental impact, energy consumption rates, and fuel costs is much more advantageous than those of other lamps, thanks to its maximum illumination power combined with minimum energy consumption, long lifespan, high efficiency, better chromatic performance, and reduced environmental impact [25,26,27]. Other applications of LED technology have been tested for the fisheries in African lakes with similar results [28,29].

In the Mediterranean Sea, PS fishing gear with the incandescent light is widely used for catching small pelagic fishes, such as the anchovy (*Engraulis encrasicolus*, Engraulidae) and the sardine (*Sardina pilchardus*, Clupeidae) [30,31,32]. These target species are highly exploited in the Adriatic Sea (central Mediterranean Sea), where the PS fleet contributes a large proportion of their catches, together with midwater pelagic pair trawlers [33,34,35,36]. To date, the use of LED technology in the Mediterranean PS fleets is not widespread and little information is reported on its catch efficiency. Some studies have been carried out on the application of LEDs on demersal fishing gears to reduce the bycatch [17,37,38], or to harvest commercial species in a lake [39]. However, an assessment of the catch efficiency and of the environmental impacts of incandescent lamps and alternative lighting technology has never been carried out.

Therefore, this study is a first attempt to investigate the catch efficiency of LED light technology compared to the traditional incandescent lamp in the Adriatic PS fishery. In particular, the performance of three different LED modules (white light, blue light, and pulsing light) was assessed using several efficiency and consumption indicators, and differences in the catch size composition were also explored. Fishery management aspects of LED performance in terms of benefits and potential risks are discussed.

## 2. Materials and Methods

### 2.1. Study Area

The Central Adriatic Sea in the Mediterranean Sea (FAO GFCM, Geographical Sub Area, GSA 17) is characterised by shallow waters not exceeding 100 m in depth, except for three depressions known as the Pomo/Jabuka Pits, ranging between 225 and 270 m in depth with a west-east progression [40]. The just-described seabed configuration allows the cold waters formed in the Northern Adriatic Sea to flow southwards and to descend into the depths of these pits, which results in ineffective water circulation and nutrient transport [41]. The area is characterised by high river inputs, which increase the productivity of the coastal waters and lower the salinity. The coasts are typically shallow and alluvial; in fact, the explored area does not exceed 200 m in depth. This area is a valuable fishing ground, where resources are mainly exploited by Italy and Croatia, with different fishing techniques, from trawling to purse seine [32,42]. In particular, year-round trawl and purse seine fisheries are conducted on anchovies and sardines by the Italian fleet in the Adriatic Sea, accounting for about 30% of the national catches [43,44]. The basin represents a relevant spawning area for the stocks of these small pelagic species [45,46,47], which are affected by changes in climate and water circulation [48,49,50].

**Figure 1:**
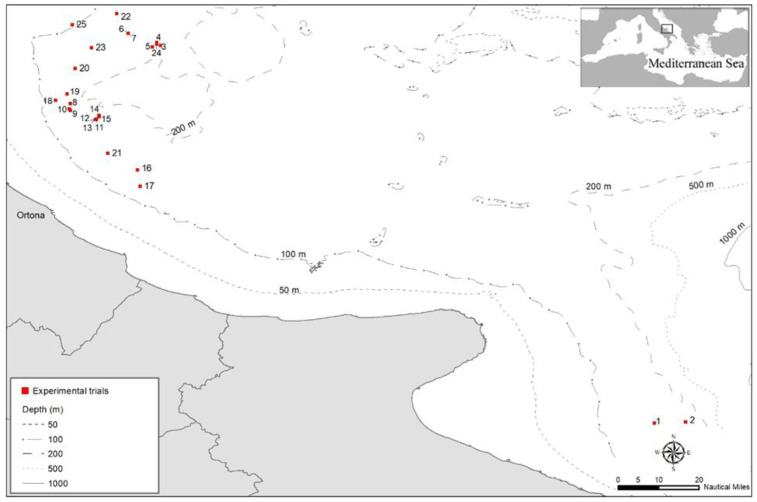
Map of the experimental trials carried out during the experimental survey from September to October 2020 in the Central and Southern Adriatic Sea (GSA 17 and 18).

The experimental survey was mainly conducted within GSA 17, an exploited area which extends to 60 miles off the Ortona Maritime Compartment, which was investigated through experimental trials (Figure 1). Only two experimental trials were carried out in the Southern Adriatic Sea (GSA 18) off the coast of the Gulf of Manfredonia because of bad weather conditions in GSA 17.

### 2.2. Features of LED Lighting Systems and the Incandescent Lamp

**Figure 2:**
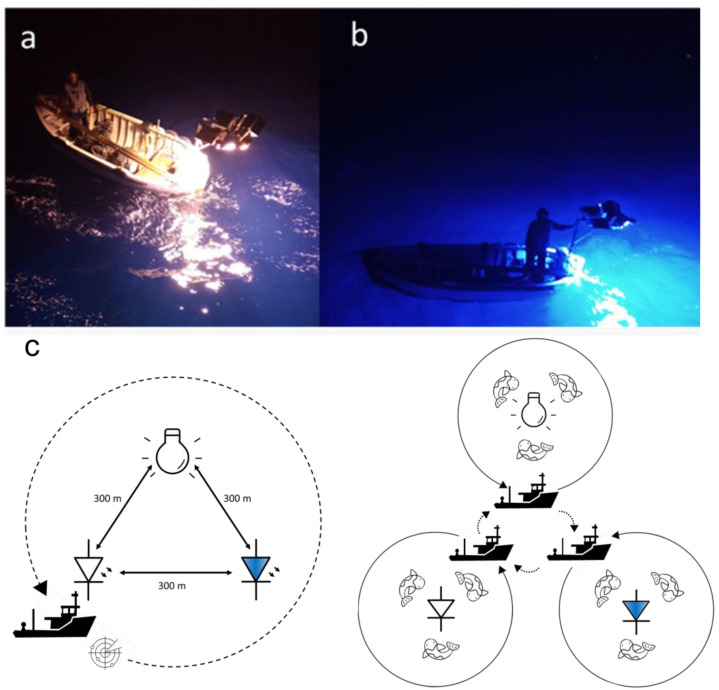
LEDs (**a**) white LED light, (**b**) blue LED light, and (**c**) outline of fishing operations conducted during experimental trials.

Three types of LED light sources were development for the field experiment: white light (LWH) with a broad spectrum perceived as white, blue light (LBLU) with an emission which peaked in the 400–500 nm range, and a pulsing white light (PULSE) (Figure 2a,b). The LED lamps were designed to achieve the following objectives: (i) the luminous flux of the LED lamps should be similar to the output flux of traditional incandescent light sources; (ii) the light sources should be powered by alternate current (AC) or direct current (DC) with a voltage of 16–42 VAC; (iii) the light sources should each have a luminous flux which quadratically increases as a function of the supplied voltage. The LED light sources were subdivided into modular elements to allow for easier manufacturing and installation. High-power LEDs based on indium gallium nitride converted by means of phosphors were used to ensure the thermal stability of the lighting system. The LWH and LBLU modules were designed in the laboratory, and each module was equipped with a microcontroller to allow the calibration of the light output and ensure functional safety features. The PULSE light source was made using LWH hardware with a firmware update allowing a switch-on time of 50 ms and a total lighting period of 100 ms. Each LWH and LBLU module had 800 W of electrical power at the maximum voltage of 42 VAC, corresponding to a current of approximately 19 A, while the PULSE module had an average power of 400 W due to its 50% duty cycle. The following configurations were installed on the small boats: LWH with 12 modules, LBLUE and PULSE both with 3 modules. Dissimilarly, the incandescent lighting system (INC) was characterised by 20 lamps with a maximum power of 1000 W each. The LWH was designed to achieve a similar luminous flux to that of the INC light sources, while the LBLU was designed to have a similar radiative flux (optical power), in the 400–500 nm range, to the LWH in its entire spectrum. In all the systems, the luminous emission was adjusted by varying the input voltage provided by the motor–generator. The electrical behaviour of the LED modules was controlled by means of the firmware installed on the microcontroller of the module; its characteristic performance followed an Ohmic law of resistance. The value of the equivalent resistance for the LED modules was set to 2 Ω to achieve a similar optical behaviour to that of the INC counterpart.

### 2.3. Experimental Survey, Data Collection, and Treatment

The experimental field survey for the comparative analysis between the LEDs and the INC lamp was carried out using a commercial PS vessel in September and October 2020, with nocturnal trials conducted from 6 p.m. until 7 a.m. the following day. The PS vessel (or mother vessel: length over all 28.46 m; gross tonnage 133 t; engine power 800 kW) carried three small boats on board, each equipped with one of the lighting systems. In addition, the mother vessel was equipped with a purse seine net with a length of 437.3 m, a height of 189 m, and a stretched mesh size of 18 mm. Regarding the use of lighting systems on the three boats, the LWH and the INC lamp were used throughout the survey, while the LBLU system was used during September, and it was replaced by the PULSE in October.

During each experimental trial, the fishing operations were characterized by several steps starting from the search for schools of fish using sonar. Once a school of fish had been identified, three small boats, each equipped with a lighting system, were released into the fishing area. The small boats were positioned with their lights on at about 300 m from each other, beginning a phase of aggregation of the school of fish attracted by the lights (Figure 2c). This distance avoided reciprocal influence between the lighting systems of the three boats. After a sufficient aggregation time of the schools under each boat, monitored with sonar by the mother vessel, the operation of encircling and capturing each school of fish began. Specifically, the mother vessel surrounded a single boat by lowering the net and hauling the catch on board. Subsequently, this operation was repeated for the other two boats concluding the experimental trial. Data on the geographic position, the depth, marine weather conditions, start and end times of each trial, the time activity of each lamp with relative voltages and the catches by species (in biomass, kg) were collected by the research personnel on board (Table 1 and Table A1). Moreover, in order to investigate the size selectivity within catches obtained by each lighting system, samples of the harvested species were collected.

**Figure 3:**
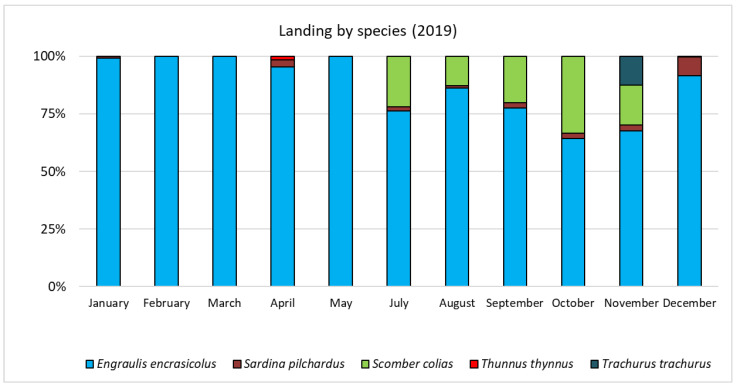
Monthly landings (%) by species in 2019 in GSA 17 obtained from the PS vessel used in the experimental survey.

In order to identify the most important target species, and to check for any anomalies in the experimental trials with respect to the usual catches by the PS vessel in the fishing area and period, monthly catch data and fishing days relative to 2019 were collected from the logbook provided by the fishermen (Table A2). In particular, anchovy was the main target species harvested in 2019, which showed a monthly landing value always above 60% of the total, with landings of approximately 44 t and 63.5 t in September and October, respectively (Figure 3). In addition, in these two months, the second important target species was the Atlantic chub mackerel (*Scomber colias*, Scombridae), with a percentage monthly landing of approximately 20–30%.

A total of 22 experimental trials were carried out during the survey, in which the LWH was used 22 times, INC 20 times, LBLU 12 times, and PULSE 5 times. These differences were determined by meteorological and sea condition variability, moon phases, the type of species targeted during the fishing trials, and the occurrence of other purse seines in the fishing area. The catch efficiency of the different lighting systems was compared on the main target species of the PS fishery; thus, only experimental trials with the occurrence of anchovy were considered, with bycatch species excluded from the analysis (Table 1). Therefore, a total of 20 trials were selected for the statistical analysis, while two trials (numbers 4 and 5) were excluded because the catches were only represented by the Atlantic chub mackerel. Specifically, individuals of this species were attracted and harvested using a yellow light obtained by using a lower voltage (20–22 V) setting of LWH light and the incandescent lamp.

### 2.4. Catch Efficiency of LED Lamps

Catch per unit effort (CPUE) was adopted to assess the performance in catch efficiency of the lighting systems, considering electric energy consumption (kilowatts per hour, kWh) and fuel consumption (litre, L) as effort units. Therefore, CPUE values (median values and interquartile ranges, IR) were calculated as kg kWh^−1^ and kg L^−1^ (Table 2).

In order to detect differences in the size and age of the anchovy specimens sampled by each lighting system, total length (TL in mm) was measured (Table A3), and the age (t) of each individual, reported as Age 0+, 1+, 2+, 3+, and 4+ (this latter also included individuals of ages older than 4), was calculated by means of the TL, adopting the Von Bertalanffy equation:

(1) $$TL=L_{\infty }\;\left(1-e^{-k\left(t-t_{0}\right)}\right)$$

where L_∞_ is the asymptotic length at which growth is zero, k is the growth rate, and t_0_ is the age at which the organisms would have had zero size. The growth parameters (L_∞_ = 18.61, K = 0.622, t_0_ = −0.849) were obtained from the stock assessment of the *E. encrasicolus* in the Adriatic Sea [51].

Length–frequency distributions of each sample obtained using the investigated lighting systems were compared using the Kolmogorov–Smirnov test (D) [52]. Outliers (identified as TL values higher than the III quartile + (1.5 × IR = interquartile range), or lower than the I quartile − (1.5 × IR), respectively) were removed before performing the D test, in order to reduce the noise in the analysis due to extreme values. In addition, the position of all catch in size with respect to the age of first maturity of the anchovy (6–8 cm, [51]) was compared. The percentage of occurrence frequency (F%) of specimens by age was calculated on the total number of sampled individuals. Differences in F% for each age class were tested using the chi-squared test (χ^2^), and a multiple comparison within and between lighting systems (post hoc analysis by U test) was applied through an absence–presence transformation (0 or 1 values, respectively) of the F% data.

Differences in CPUE values between experimental LED lamps and the traditional incandescent lamp were compared using the Kruskal–Wallis test (KW) and a nonparametric post hoc test, based on the Bonferroni-corrected pairwise Mann–Whitney test (U) [53]. This choice was due to the non-normal distribution of the data tested by the Shapiro–Wilk test (Table A4) [54]. The statistical analysis was carried out using PAST 4.03 [55].

### 2.5. Energy Consumption and CO_2_ Emissions

The electrical energy consumed by each LED module and the INC lamp was calculated using the electric power generated during the trial by each motor–generator on board the boats according to the equation:

(2) $$P\left(\mathrm{Watt}\right)=\frac{V^{2}}{R}\times n.\mathrm{LED}\;\mathrm{modules}\;\mathrm{or}\;\mathrm{INC}\;\mathrm{lamps}$$

where the voltage (V) is measured on board, while the equivalent electric resistance (R) was equal to 2.2 Ω and 1.55 Ω for LED modules and INC lamps, respectively. Thus, the total electric consumption (in kWh) of each lighting system was calculated as the product of the electric power multiplied by the time of activity of each LED module or INC lamp (Table A5). The fuel consumption (diesel in L) was calculated for each tested lighting system as:

(3) $$\mathrm{Fuel}=\frac{\left(\mathrm{Electric}\;\mathrm{consumption}\times 240\right)}{\mathrm{fuel}\;\mathrm{density}\;}$$

where electric consumption is expressed in kWh and the diesel density is equal to 850 gr L^−1^ (Table A5). In addition, comparisons between the consumption of each lamp in terms of electric energy consumption (kWh) and hourly fuel consumption (L h^−1^) were carried out, as the CO_2_ emissions generated by the fuel combustion during the fishing activity were also estimated (Table 2). According to the EPA (United States Environmental Protection Agency, https://www.epa.gov/, accessed on 25 September 2021) [56], 1 L of diesel (of density of 835 gr L^−1^, [57]) contains 720 gr (86.2%) of carbon requiring 1920 gr of oxygen for its combustion (1:2 stoichiometric ratio). Therefore, approximately 2.64 kg of CO_2_ is released into the atmosphere by every 1 L of diesel burnt.

Finally, an estimate of the economic costs of each lighting system during fishing activities was assessed using hourly fuel consumption with three different fuel prices (Euro per litre, EURL^−1^): average price during the survey period (EUR 0.29 L^−1^, [58]) (Table A5).

Differences in electric energy consumed, hourly fuel consumption, CO_2_ emissions, and economic costs between experimental LED lamps and the traditional incandescent lamp were compared using the same statistical tests applied to the CPUE analysis (Table A5).

## 3. Results

### 3.1. Catch Efficiency of LED Lamps

Experimental catches, obtained from 22 trials, were characterized by high abundances of anchovies, which represent 66% and 54% of total catches with LWH and INC, respectively. *S. colias*, *S. pilchardus*, the shortfin squid (*Illexcoindetii*, Ommastrephidae), the swordfish (*Xiphias gladius*, Xiphiidae), the Atlantic bonito (*Sarda sarda*, Scombridae), and the horse mackerel (*Trachurus mediterraneus*, Carangidae) were the main bycatch species detected in the survey (Table 1).

Considering the 20 experimental trials selected for the comparison analysis, the highest time activity of the lighting systems was detected for the LWH (68 h), followed by the INC (38 h), the LBLU (32 h), and the PULSE (16 h) (Table A1). In addition, the electrical energy consumption showed the highest value for the INC (1023 kWh), followed by the LWH (649 kWh), the PULSE (99 kWh), and the LBLU (81 kWh) (Table A5). Moreover, the highest total fuel consumption was detected for the INC (339 L), and then the LWH (181 L), the PULSE (28 L), and the LBLU (26 L). The highest anchovy catch was observed for the LWH (39.1 t), followed by the INC (22.2 t), and the LBLU and the PULSE, both with about 17.0 t. Among these 20 trials, anchovy catches corresponded to about 98% of the total for each lighting system (Table 1).

**Figure 4:**
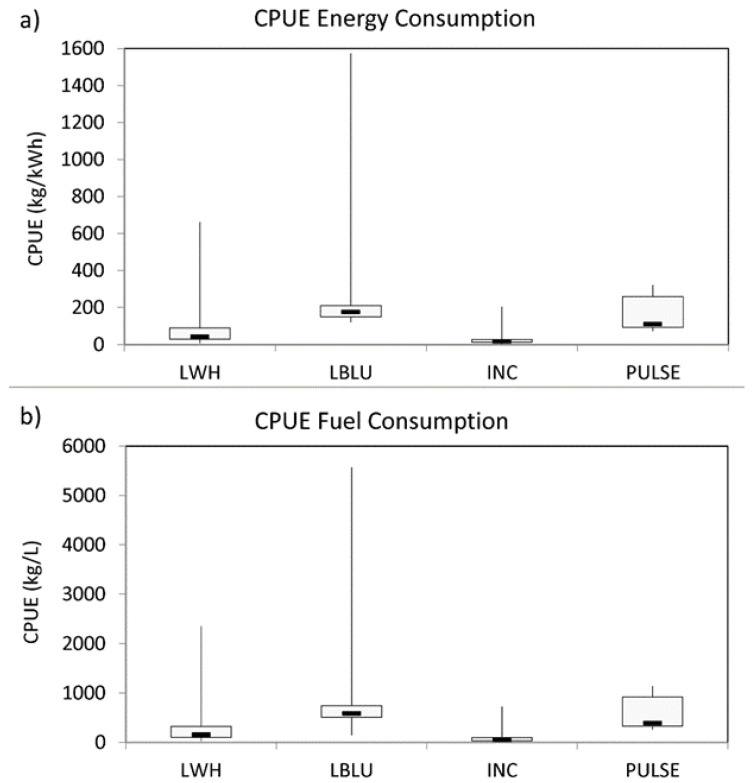
Catch per unit effort (CPUE) calculated in (**a**) energy consumed (kg kWh^−1^) and (**b**) fuel consumption (kg fuel L^−1^) for each LED tested (White, LWH; Blue, LBLU; Pulsing, PULSE) and incandescent lamp (INC).

Considering the catch efficiency, the CPUE per energy consumption of LBLU (median value of 177 kg kWh^−1^, IR = 59.60) was significantly higher than that of INC (median value of 18 kg kWh^−1^, IR = 15.30; U = 7, *p* < 0.001) and LWH (median value of 42 kg kWh^−1^, IR = 62.04; U = 19, *p* < 0.001) (Figure 4a and Table A6). Moreover, the PULSE (median value of 111 kg kWh^−1^, IR = 165.44) together with LWH proved to be significantly more efficient than INC (U = 3, *p* < 0,05; U = 75, *p* < 0.05, respectively). Similar differences were observed for the CPUE per fuel consumption, where that of LBLU (median value of 593 kg L^−1^, IR = 233.48) was significantly higher than INC (median value of 62 kg L^−1^, IR = 70.80; U = 9, *p* < 0.001) and LWH (median value of 159 kg L^−1^, IR = 219.73; U = 30, *p* < 0.01) (Figure 4b and Table A6). In addition, the median CPUE of INC was significantly lower than LWH (U = 70, *p* < 0.01) and PULSE (median value of 392 kg L^−1^, IR = 585.93; U = 3, *p* < 0.05).

**Figure 5:**
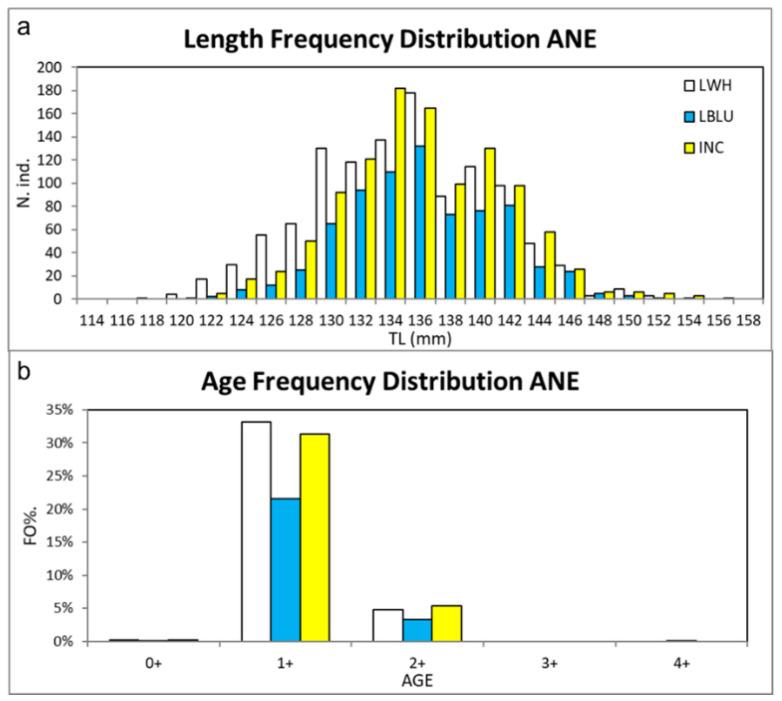
(**a**) Length–frequency distribution (LFD) of the total length (TL in mm) and (**b**) age frequency distribution calculated for the anchovies (ANE) sampled using LWH, LBLU, and INC. Outliers of TL values, out of the range 114–159 mm, were excluded from the plot.

All anchovy samples (Tot. N. ind. = 2969) obtained using the lighting systems showed the same median TL (136 mm), while the interquartile range of the LWH (IR = 9 mm) was wider than LBLU and the INC lamps (both IR = 7 mm) (Table A3). The min–max TL ranges calculated for the LWH, the LBLU, and the INC lamp were equal to 80–153 mm, 93–175 mm, and 83–156 mm, respectively. All sampled specimens showed the minimum TL value as higher than the size at first maturity. A total of 2957 specimens (LWH N. ind. = 1128; LBLU N. ind. = 740; INC N. ind. = 1089) were selected for the statistical analysis on length–frequency distributions, excluding outliers. The length–frequency distribution of anchovy collected using LWH resulted in significantly different values from those using LBLU (D = 0.116, *p* < 0.001) and INC (D = 0.094, *p* < 0.001), while no difference was observed between LBLU and INC (Figure 5a and Table A6).

Age 1+ anchovies (N. ind. = 2555) were the most frequent specimens by significant amounts in all lighting systems, representing over 85% of the total (*p* < 0.05), while specimens of Age 2+ (N. ind. = 402) accounted for 14% of the total (Figure 5b and Table A7). No significant difference in F% was observed between Age 1+ anchovies sampled by the LWH (N. ind. = 985, 33%), the LBLU (N. ind. = 641, 22%), or the INC (N. ind. = 929, 31%).

### 3.2. Energy Consumption and CO_2_ Emissions

**Figure 6:**
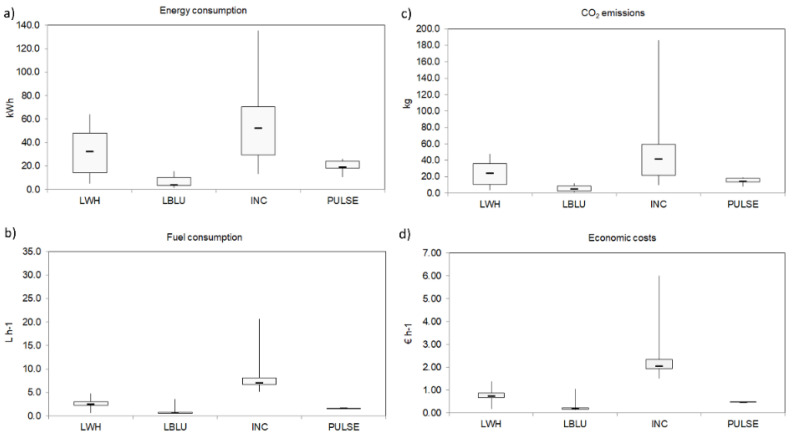
Consumption in terms of (**a**) energy (kWh), (**b**) hourly fuel (L h^−1^), (**c**) CO_2_ emissions (kg), and (**d**) economic costs (EURh^−1^) for each tested LEDs (White, LWH; Blue, LBLU; Pulsing, PULSE) and the incandescent lamp (INC).

The comparative analysis of hourly fuel consumption showed that the highest median value was measured for INC (7.1 L h^−1^, IR = 1.40), which was significantly higher than LWH (2.5 L h^−1^, IR = 0.66; U = 0, *p* < 0.001), LBLU (0.7 L h^−1^, IR = 0.21; U = 0, *p* < 0.001), and PULSE (1.7 L h^−1^, IR = 0.04; U = 0; *p* < 0.01) (Figure 6b, Table A6). Concerning CO_2_ emissions estimated from fuel consumption, the highest median value was observed for INC (41.4 kg, IR = 37.33) followed by LWH (24.1 kg, IR = 25.07), PULSE (14.2 kg, IR = 4.65), and LBLU (4.9 kg, IR = 6.14) (Figure 6c and Table A6). The latter value was significantly lower than for the other lighting systems (*p* < 0.05; Table A6).

According to the average fuel price during the survey period, the highest significant hourly cost (median value 2.05 EURh^−1^) was estimated for the INC (*p* < 0.01) (Figure 6d and Table A6). In addition, the LWH showed a median hourly cost of 0.74 EURh^−1^, which was significantly higher than for the LBLU (0.20 EURh^−1^, U = 23; *p* < 0.01) and the PULSE (0.48 EURh^−1^, U = 10; *p* < 0.05). Thus, the reduction in costs obtained by using LED systems compared to an incandescent lamp was 64% and 90% for both LWH and LBLUE, respectively (Table A5).

## 4. Discussion

The route towards sustainable fisheries management requires an effort in the adoption of the ecosystem-based fishery management approach (EBFM), which is a focal point in the achievement of Goal 14 “Life Below Water” of the Sustainable Development Goals of the United Nations [5]. The holistic approach should be strongly considered in the assessment of the technological advancements in the fishery, focusing on the positive and negative effects induced by catch efficiency changes [13]. Indeed, the temporal increase in catch efficiency (usually indicated as catchability or nominal effort) is not often properly considered in fishery management, with the consequence of making technological innovations applied to reduce the fishing pressure on resources and marine ecosystems ineffective [8,9]. In this regard, this study tried to assess the performance of LED technology in the purse seine fishery, providing some indications on the catch efficiency and environmental impacts derived from this innovative fishing device. No less importantly, two of the tested LEDs (blue and pulsing LEDs) were experimented with for the first time in the anchovy fishery, the main commercial species in the Adriatic Sea (central Mediterranean Sea). This choice follows the hypothesis that different-coloured lights could have a greater attraction power for several species, such as blue light [17], or a different setting of lighting systems could reduce both energy and fuel consumption, such as in the case of the pulsing white LED. In addition, the catch quantity and its composition in species observed during the experimental survey are consistent with the landing data from the previous year, validating the procedure adopted in the experimental design and the results.

### 4.1. Catch Efficiency of LED Lamps and Biological Aspects

The results obtained during the survey confirm the possibility of obtaining consistent benefits using the LED lighting systems in terms of yields, which are increased compared to the traditional incandescent lamp. Concerning the catch efficiency of the LED lights, the white LED, which emits light in the same light spectra as the incandescent lamp, increased the yield by over 2 times per consumption unit of energy and fuel. This result is very similar to the estimations reported at the global scale for LED technology applied to the purse seine fishery [17]. It seems likely that the illumination zone produced around the boats by LEDs was more concentrated in a specific direction, increasing the efficiency of attraction, as observed by Nguyen and Tran [22]. Moreover, the yield efficiency increased by values of up to approximately 6 times with the use of the pulsing white LED, as measured using both consumption units. This increase was due to the energy savings resulting from the flashing of the LED. Therefore, this setting could be an interesting improvement in the performance, but further studies are required to understand the effective benefits of this LED light configuration. The highest catch efficiency was estimated for the blue LED, which showed yields of over 9 times higher than the incandescent lamp. This higher efficiency of LEDs could be explained by the increase in the concentration of light in the illumination area [22], and by the effect of different wavelengths. Indeed, the greater attraction of blue light (wavelength 465 nm), due to its higher water penetration [59], has also been observed for some rockfish species (*Sebastes* spp.) in Japanese waters [60] and for the fishery of the snow crab (*Chionoecetes opilio*) in the province of Newfoundland and Labrador, Canada [61]. Further studies should be carried out to quantify the attraction effects on different species and the effective increase in the light penetration in water. In addition, results obtained from the analysis of length–frequency distributions highlighted similar performances in the size of anchovies selected by the LED systems and by the incandescent lamp, with the same median total length value equal to 13.6 cm. Remarkably, these values of total length and the interquartile ranges were abundantly higher than the first maturity size of anchovy in the Adriatic Sea, indicating the absence of impacts of LED systems on immature specimens (TL < 8 cm, [51]). A slight difference was observed between the LFD of the LWH and those of other systems, where the former LED seemed to catch a higher number of small specimens (TL range 120–130 mm) than other systems. A possible explanation could still be linked to the higher water penetration of LBLU and the distribution pattern of the anchovy juveniles and adults along the bathymetric gradient. In fact, the depth is an important factor for the habitat selection and Giannoulaki et al. [62] reported a wider movement of the adults towards deep habitats up to 180 m in depth in late autumn, while juveniles were distributed in shallower waters. Moreover, a similar distribution pattern of juveniles and adults along the depths has been observed in the Bay of Biscay, where within the juvenile component, the smaller specimens also tended to be aggregated near the surface, while larger specimens were located at greater depths [63]. However, further experiments should be performed collecting more samples to assess the effective size selectivity of LED systems and exploring potential influences of seasonality on migrating species [64], as well as other environmental factors acting on different life stages of the anchovy [65].

### 4.2. Energy Consumption and CO_2_ Emissions: Benefits and Critical Points

Estimates obtained for hourly fuel consumption and CO_2_ emissions stressed the potential advantages derived from using LEDs in the reduction of the carbon footprint due to the PS fishery. Remarkably, CO_2_ emission estimates for the incandescent lamp were 1.7 and 2.9 times higher than those for the white and pulsing LED, respectively. In addition, the incandescent lamp emitted GHG amounts over 8 times greater than the blue LED, which again showed the best performances. From these outlines it can be estimated that a PS vessel, working with a traditional gear configuration in the Adriatic Sea, generates an average of 5 t of CO_2_ per year during the fishing operations using an incandescent lamp, while emissions could decrease below 3500 t per year by adopting the white LED. These estimates could be used to develop scenarios on the GHG emissions of the overall fleet operating in the study area, matching the information related to the nominal fishing effort [59]. Indeed, global analysis on GHG emissions generated by world fisheries indicated that a reduction in the fishery’s carbon footprint depends on several factors, among which are the increase in catches, the fishing capacity, and effort [66]. The evaluation of the economic costs highlighted the positive impacts derived from LED technology on the operational costs of the PS fishery, with the fuel cost-saving percentages all being higher than 60%. This estimated value for LWH (64%) in our experiment was very similar to those obtained in experiments conducted between LED and fluorescent lamps (67% savings) in Vietnam [22] and between LED and metal halide lamps (64% savings) in the PS fleet of Indonesia [20]. However, a complete economic assessment should be performed considering device maintenance and fixed costs, to identify when the financial breaking point could be reached, thus obtaining profits for the fishing enterprise [67].

Although LED technology provides clear economic and environmental benefits, there are some issues regarding the exploitation of fishery stocks that should be further explored in the evaluation of LED performance. Indeed, the high catch efficiency could induce an increase in the fishing mortality of these fishing resources. Consequently, an unsustainable fishing pressure could affect small pelagic stocks, which are considered in a condition of overexploitation in the Adriatic basin [35,51]. In particular, the increase in catch efficiency due to technological advancements should be managed, considering the nominal effort (or other factors) to balance possible higher fishing pressure [13]. To date, regulations on the power of lights have been adopted in the Spanish purse seine fleet, with limitations of the maximum luminous intensity to 100,000 lumens, as well as requests for replacement of those lights with low energy consumption systems [68]. Therefore, the application of such regulations should take into account both the knowledge of the characteristics of purse seining in the Adriatic Sea and the management strategies of the small pelagic stocks that can best be adapted to achieve sustainable exploitation [69].

## 5. Conclusions

In conclusion, the LED technologies developed and applied in this study clearly show potential benefits at the economic level for the fishermen and the possibility of mitigating indirect negative effects on the environment due to fuel combustion and GHG emissions. Although the results obtained showed clear benefits, the application of new technology to improve the catch efficiency of fishing gears should be carefully considered, with particular attention being paid to a key element in the sustainability of the fisheries [70]. The lack of regulation of fishing activities, as well as the absence of technologically advanced control in fishery management plans, could cause unwanted long-term effects. Therefore, the introduction of innovative fishing devices with a resultant increase in catch-efficiency performance can be effectively realized by adopting the EBFM approach to sustainability.

## Figures and Tables

**Table 1:** Total catches (kg) by species from 22 experimental trials (Tr.) obtained using all light systems: white LED (LWH), blue (LBLU), incandescent lamp (INC), and pulsing LED (PULSE). Species are indicated by FAO code: *E. encrasicolus* (ANE), *S. colias* (MAS), *Illexcoindetii* (SQM), *S. pilchardus* (PIL), *Xiphias gladius* (SWO), *Sarda sarda* (BON), and *Trachurus mediterraneus* (HMM).

Tr.	Species	LWH	LBLU	INC	Tr.	Species	LWH	LBLU	INC	PULSE
1	ANE	474		316	15	ANE	586	654	559	
1	MAS	129		87	15	MAS	6	7	5	
1	SQM	3		3	15	PIL	6	6	6	
2	MAS	112		73	15	SQM	6	6	5	
2	ANE	434		252	16	ANE	700	1681	420	
2	SQM	14		7	16	MAS	17	41	10	
3	ANE	755		247	16	SQM	2	4	1	
3	MAS	7		2	17	ANE	283	420	274	
3	SQM	7		3	17	MAS	7	9	6	
4	MAS	13928		13,321	18	ANE	2100		560	1400
5	MAS	5438		5185	18	MAS	7		2	2
6	ANE	6478	3239	3220	18	SQM	7		2	2
6	SWO	73			19	ANE	5250		1960	8400
7	ANE	3543	2814	2716	19	SQM	7		3	11
8	ANE	1910	1751	1645	20	ANE	2002		3003	2695
8	MAS	41.1	37.6	35.4	21	ANE	1600			1700
8	PIL	3.9	3.7	3.4	21	MAS	280			284
8	HMM	0.261	0.237	0.27	22	ANE	2880			2800
8	BON	0.012			22	MAS	115			119
9	ANE	654	598	617						
10	ANE	732	520	674		Total Catch (kg)	59,382.3	17,145.5	40,987.1	17,413.0
11	ANE	5240	2096	3144		Catch (%)	44	13	30	13
12	ANE	786	687	491		Mean	1263.5	714.4	999.7	1741.3
13	ANE	901	501	582		Stand. Error	365.39	201.38	359.96	818.45
14	ANE	1798	1995	1495		ANE Tot. Catch (kg)	39,106	16,956	22,175	16,975
14	MAS	27	31	23		ANE Tot. Catch (%)	98	99	99	98
14	PIL	17	23	17						
14	SQM	16	21	12						

**Table 2:** CPUE (energy, kg kWh^−1^, and fuel consumption, kg L^−1^), CO_2_ emissions (kg), and hourly fuel consumption (L h^−1^) by LWH, LBLU, PULSE, and INC estimated by 20 experimental trials (Tr.) used in the statistical analysis.

	CPUE Energy				CPUE Fuel				CO_2_ Emissions				Hourly Fuel Consumption			
Tr.	LWH	LBLU	INC	PULSE	LWH	LBLU	INC	PULSE	LWH	LBLU	INC	PULSE	LWH	LBLU	INC	PULSE
1	8.37		4.22		29.63		14.93		42.2		55.9		3.6		7.1	
2	13.28		4.82		47.02		17.07		24.4		39.0		3.1		7.4	
3	23.73		4.20		83.98		14.88		23.7		43.8		2.4		7.4	
6	101.47	207.88	23.80		359.38	736.24	84.28		47.6	11.6	100.9		2.8	0.8	7.1	
7	662.41	1573.73	204.56		2346.36	5573.61	724.48		4.0	1.3	9.9		2.3	0.8	6.4	
8	41.19	149.58	23.37		163.16	529.75	23.37		30.9	8.7	185.9		2.5	0.7	19.4	
9	45.39	204.68	21.97		223.84	146.98	77.81		7.7	10.7	20.9		1.5	3.6	15.3	
10	43.66	150.29	18.40		154.62	532.29	65.15		12.5	2.6	27.3		2.6	0.5	20.7	
11	111.44	215.77	31.74		394.68	764.17	112.43		35.1	7.2	73.8		2.4	0.6	6.7	
12	58.96	215.22	16.78		208.86	763.33	59.44		9.9	2.4	21.8		4.8	0.7	14.6	
13	83.37	126.84	29.42		295.28	448.91	104.30		8.1	2.9	14.7		2.2	0.8	6.7	
14	35.31	170.08	13.71		125.06	602.35	48.54		38.0	8.7	81.3		2.8	0.7	8.3	
15	30.01	164.31	18.39		106.26	583.93	65.14		14.6	3.0	22.7		3.2	0.6	7.1	
16	16.47	183.61	5.17		58.34	650.30	18.32		31.7	6.8	60.5		3.0	0.6	7.1	
17	19.87	120.69	16.45		70.37	427.44	58.27		10.6	2.6	12.4		4.3	0.6	7.1	
18	40.14		12.39	73.61	142.15		43.88	260.69	39.0		33.7	14.2	2.6		5.2	1.64
19	86.81		31.51	321.10	307.44		111.60	1137.23	45.1		46.4	19.5	2.2		5.5	1.60
20	139.03		49.88	110.63	492.39		176.67	391.82	10.7		44.9	18.2	0.6		5.6	1.68
21	40.16			93.82	142.24			332.28	29.7			13.5	2.3			1.69
22	184.62			259.26	653.85			918.21	11.6			8.1	2.4			1.66
Median	42	177	18	111	159	593	62	392	29.1	5.9	50.0	17.1	2.9	0.7	7.7	1.7
IR	62.04	59.60	15.30	165.44	219.73	233.48	70.80	585.93	38.41	8.94	59.75	12.21	0.81	0.18	4.50	0.00
Min	8	121	4	74	30	147	15	261	12.9	3.1	26.6	16.3	0.6	0.6	5.6	1.7
Max	662	1574	205	321	2346	5574	724	1137	57.5	14.0	224.6	23.6	8.0	3.8	34.5	2.0

## Data Availability

Data are available in the final report of the Se.Le.Ca project: Carlucci R., Trivellin N., Orlandi V.T., Meneghesso G., Meliadò E., Cipriano G., Cascione D., Rositani L., Tursi A., Capezzuto F., Piva F., Pizzolato A., Bolognese F., Lombardi G., Ricci P. (2021). Se.Le.Ca.: Selective LED Catch and Antimicrobial blue light treatment. Relazione tecnica, 95 pp. Programma PO FEAMP 2014/2020 mis. 1.26 cod. 0002/INP/17.

## Appendix A

**Table A1:** Data collected in experimental trials (Tr.): date, latitude (Lat), and longitude (Long), depth (in m), the start time of boats release (R. start time), start and end fishing time, and the time activity (TA in hours minutes) for each LED tested (white, LWH; blue, LBLU; pulsing, PULSE) and the incandescent lamp (INC).

Tr.	Date	Lat	Long	Depth (m)	R. Start Time	F. Start Time	F. End Time	TA LWH (h.m)	TA LBLU (h.m)	TA INC (h.m)	TA PULSE (h.m)
1	06/09/20	41°28,906	016°53,428	117	20:18	00:25	00:42	4.24		3.00	
2	07/09/20	41°29,160	017°01,140	143	20:23	23:06	23:24	3.01		2.00	
3	10/09/20	42°62,104	014°51,525	110	00:05	03:25	03:47	3.42		2.15	
4	14/09/20	43°02,843	014°50,629	114	20:13	21:08	21:23	1.00		0.30	
5	14/09/20	43°01,701	014°49,559	110	01:20	02:08	02:26	0.38		0.19	
6	15/09/20	43°05,115	014°43,587	515	20:45	03:41	03:58	6.32	5.38	5.25	
7	15/09/20	43°05,120	014°43,590	115	04:20	04:55	05:20	0.39	0.36	0.35	
8	16/09/20	42°47,711	014°29,308	123	20:51	01:58	02:14	4.39	4.50	3.38	
9	17/09/20	42°46,102	014°29,301	121	02:25	03:30	03:55	1.55	1.08	0.31	
10	17/09/20	42°46,444	014°29,095	121	04:02	05:56	06:10	1.50	1.55	0.30	
11	20/09/20	42°43,946	014°35,706	149	21:17	02:35	02:52	5.35	4.56	4.11	
12	21/09/20	42°43,940	014°35,688	150	03:12	04:15	04:27	0.47	1.15	0.34	
13	21/09/20	42°43,830	014°35,522	148	04:45	06:12	06:25	1.22	1.24	0.50	
14	21/09/20	42°44,856	014°36,340	155	22:50	03:52	04:10	5.03	4.39	3.42	
15	22/09/20	42°44,558	014°36,470	149	04:30	06:20	06:34	1.43	1.56	1.13	
16	22/09/20	42°31,345	014°45,900	138	22:45	02:50	03:05	4.03	4.20	3.15	
17	22/09/20	42°27,316	014°46,533	117	04:15	05:45	06:02	0.56	1.47	0.40	
18	18/10/20	42°48,570	014°25,680	129	21:30	02:30	03:15	5.45		2.26	3.17
19	18/10/20	42°50,105	014°28,503	114	04:15	05:30	06:45	7.50		3.11	4.36
20	19/10/20	42°56,422	014°30,508	115	21:40	05:15	06:30	6.30		3.01	4.06
21	20/10/20	42°35,507	014°38,600	107	21:30	02:30	03:30	4.50			3.02
22	21/10/20	43°09,930	014°40,745	97	23:10	03:40	04:00	1.50			1.50

**Table A2:** Monthly landing (kg) by species and fishing days in 2019 of the experimental PS vessel in the GSA 17–18. Species are indicated by FAO code: *E. encrasicolus* (ANE), *S. colias* (MAS), *I. coindetii* (SQM), *S pilchardus* (PIL), *X. gladius* (SWO), *T. thynnus* (BFT), and *T. mediterraneus* (HMM). In June, the PS vessel operated in the GSA 19, thus data are not reported.

Month	ANE	PIL	MAS	BFT	HMM	SWO	Fishing Days
January	27,440	231					6
February	15,642						4
March	1084						1
April	49,875	1575		782		33	10
May	25,900					40	5
July	38,534	924	11,134				14
August	53,605	770	7861			64	16
September	44,065	1264	11,515				12
October	63,510	2310	33,080			85	18
November	13,160	468	3360		2450		6
December	20,090	1750	70				7
Total	35,2905	9292	67,020	782	2450	222	99

**Table A3:** Number of specimens by class size in total length (TL in mm) measured for each sample obtained using lighting systems (left side); and age, expressed as number of individuals and % estimated from TL. Main statistics were calculated to identify TL outliers (see the main text for details).

TL	LWH	LBLU	INC
80	2	0	0
82	1	0	1
84	0	0	0
86	1	0	0
88	0	0	1
90	1	0	0
92	0	1	1
94	0	0	1
96	0	0	0
98	0	0	1
118	1	0	0
120	4	0	1
122	17	2	5
124	30	8	17
126	55	12	24
128	65	25	50
130	130	65	92
132	118	94	121
134	137	110	182
136	178	132	165
138	89	73	99
140	114	76	130
142	98	81	98
144	48	28	58
146	29	24	26
148	3	5	6
150	9	3	6
152	3	1	5
154	0	1	3
156	0	0	1
174	0	1	0
Main statistics and TL outliers			
	**LWH**	**LBLU**	**INC**
Total N. individuals	1133	742	1094
Min	80	93	83
Max	153	175	156
Median	136	136	136
I quartile	131	133	133
III quartile	140	140	140
IR	9	7	7
Outliers upper limit (TL mm)	159	154	154
Outliers lower limit (TL mm)	114	119	119
**AGE**	**LWH**	**LBLU**	**INC**
Age 0+ N. (%)	5 (0.2%)	1 (<0.1%)	5 (0.2%)
Age 1+ N. (%)	985 (33.2%)	641 (21.6%)	929 (31.2%)
Age 2+ N. (%)	143 (4.8%)	99 (3.3%)	160 (5.4%)
Age 3+ N. (%)	-	-	-
Age 4+ N. (%)	-	1(<0.1%)	-

**Table A4:** Normality test (Shapiro–Wilk, W) applied to CPUEs, energy, and hourly fuel consumptions, CO_2_ emissions compared among each lighting system, and the length–frequency distribution (LFD) of anchovy obtained by the LWH, LBLU, and INC. Significant *p* values (<0.05) in red indicate a non-normal distribution of data.

Normality Test: Shapiro–Wilk (W)	LWH	LBLU	INC	PULSE
N. samples	20	12	18	5
CPUE Energy, W:	0.507	0.403	0.483	0.846
*p* value	<0.001	<0.01	<0.001	0.1818
CPUE Fuel, W:	0.511	0.439	0.485	0.846
*p* value	<0.001	<0.01	<0.001	0.1818
Electric Energy Consumption, W:	0.905	0.8484	0.9419	0.9425
*p* value	0.050	<0.05	0.312	0.684
Hourly Fuel Consumption, W:	0.926	0.431	0.697	0.943
*p* value	0.1282	5.71E−03	7.42E−02	0.685
CO_2_ Emissions, W:	0.911	0.877	0.789	0.942
*p* value	0.066	0.081	<0.01	0.682
Economic Costs, W:	0.925	0.443	0.695	0.821
*p* value	0.121	<0.001	<0.001	0.119
**LFD**	**LWH**	**LBLU**	**INC**	-
N. samples	1128	739	1086	-
LFD LWH sample, W	0.9946	0.9931	0.9944	-
*p* value	<0.001	<0.01	<0.001	-

**Table A5:** Energy, fuel consumption, and hourly costs of each lighting system tested in the 20 experimental trials (Tr.) selected for the statistical analysis.

	Energy Consumption (kWh)				Fuel Consumption (L)				Economic Costs (EUR h^−1^)			
Tr.	LWH	LBLU	INC	PULSE	LWH	LBLU	INC	PULSE	LWH	LBLU	INC	PULSE
1	56.7		74.9		16.0		21.2		1.05		2.05	
2	32.7		52.3		9.2		14.8		0.89		2.14	
3	31.8		58.8		9.0		16.6		0.70		2.14	
6	63.8	15.6	135.3		18.0	4.4	38.2		0.80	0.23	2.05	
7	5.3	1.8	13.3		1.5	0.5	3.7		0.67	0.24	1.86	
8	46.4	11.7	70.4		11.7	3.3	70.4		0.73	0.20	5.62	
9	14.4	2.9	28.1		2.9	4.1	7.9		0.44	1.04	4.45	
10	16.8	3.5	36.6		4.7	1.0	10.3		0.75	0.15	6.00	
11	47.0	9.7	99.0		13.3	2.7	28.0		0.69	0.16	1.94	
12	13.3	3.2	29.3		3.8	0.9	8.3		1.39	0.21	4.23	
13	10.8	4.0	19.8		3.1	1.1	5.6		0.65	0.23	1.94	
14	50.9	11.7	109.1		14.4	3.3	30.8		0.83	0.21	2.41	
15	19.5	4.0	30.4		5.5	1.1	8.6		0.93	0.17	2.05	
16	42.5	9.2	81.2		12.0	2.6	22.9		0.86	0.17	2.05	
17	14.2	3.5	16.7		4.0	1.0	4.7		1.25	0.16	2.05	
18	52.3		45.2	19.0	14.8		12.8	5.4	0.75		1.52	0.47
19	60.5		62.2	26.2	17.1		17.6	7.4	0.63		1.60	0.47
20	14.4		60.2	24.4	4.1		17.0	6.9	0.18		1.63	0.49
21	39.8			18.1	11.2			5.1	0.67			0.49
22	15.6			10.8	4.4			3.0	0.70			0.48
Median	32.2	4.0	55.5	19.0	9.1	1.9	15.7	5.4	0.74	0.20	2.05	0.48
IR	33.58	6.82	44.25	6.24	9.5	2.3	14.1	1.8				
Min	5.3	1.8	13.3	10.8	1.5	0.5	3.7	3.0	0.18	0.15	1.52	0.47
Max	63.8	15.6	135.3	26.2	18.0	4.4	70.4	7.4	1.39	1.04	6.00	0.49
Reduction costs vs. INC (median value)									−1.31	−1.84	-	−1.56
Reduction costs vs. INC (%)									64	90		76

**Table A6:** Mann–Whitney test (U) applied to the CPUEs (kg kWh^−1^ and kg L^−1^), energy consumption (kWh), hourly fuel consumption (L h^−1^), CO_2_ emissions (kg), and economic costs (EUR h^−1^) of all lighting systems. Kolmogorov–Smirnov test (D) applied to the length–frequency distribution (LFD) of anchovy sampled by LWH, LBLU, and INC systems. U/D values and *p* values are below and above the diagonal of each table, respectively. Significant *p* values are indicated in red.

U Test									
**CPUE Energy Consumption**	LWH	LBLU	INC	PULSE	**CPUE Fuel Consumption**	LWH	LBLU	INC	PULSE
LWH	-	<0.001	<0.05	0.194	LWH	-	<0.01	<0.01	0.194
LBLU	19	-	<0.001	1	LBLU	30	-	<0.001	1
INC	75	7	-	<0.05	INC	70	9	-	<0.05
PULSE	18	22	3	-	PULSE	18	25	3	-
**Energy Consumption**	LWH	LBLU	INC	PULSE	**Hourly Fuel Consumption**	LWH	LBLU	INC	PULSE
LWH	-	<0.001	0.1207	1	LWH	-	<0.01	<0.001	<0.05
LBLU	12	-	<0.001	<0.05	LBLU	25.5	-	<0.001	0.054
INC	100	1	-	0.061	INC	0	0	-	<0.01
PULSE	35	3	10	-	PULSE	10	5	0	-
**CO_2_ Emissions**	LWH	LBLU	INC	PULSE	**Economic Costs**	LWH	LBLU	INC	PULSE
LWH	-	<0.001	0.095	1.00	LWH		<0.01	<0.001	<0.05
LBLU	20	-	<0.05	<0.05	LBLU	23		<0.001	0.057
INC	97	2	-	0.061	INC	0	0		<0.05
PULSE	36.5	4	10	-	PULSE	10	5	0	
**D Test**									
**LFD**	LWH	LBLU	INC						
N. samples	1128	739	1086						
LWH	-	<0.001	<0.001						
LBLU	0.116	-	0.869						
INC	0.094	0.032	-						

**Table A7:** U test applied to the age–frequency distribution of anchovies for LWH, LBLU, and INC systems. U values and *p* values are below and above the diagonal of each table, respectively. Significant *p* values (<0.05) are in red.

		LWH			LBLU			INC		
		Age 0+	Age 1+	Age 2+	Age 0+	Age 1+	Age 2+	Age 0+	Age 1+	Age 2+
LWH	Age 0+		<0.001	0.870	1.000	<0.001	1.000	1.000	<0.001	0.870
	Age 1+	3350		<0.001	<0.001	1.000	<0.001	<0.001	1.000	<0.001
	Age 2+	4750	3600		0.870	<0.05	1.000	0.870	<0.001	1.000
LBLU	Age 0+	4750	3350	4750		<0.001	1.000	1.000	<0.001	0.870
	Age 1+	3900	4450	4150	3900		<0.01	<0.001	1.000	<0.05
	Age 2+	4850	3500	4900	4850	4050		1.000	<0.001	1.000
INC	Age 0+	4850	3350	4750	4850	3900	4850		<0.001	0.870
	Age 1+	3450	4900	3700	3450	4550	3600	3450		<0.001
	Age 2+	4750	3600	5000	4750	4150	4900	4750	3700	
